# Pituitary adenylate cyclase-activating polypeptide expression in peripheral blood mononuclear cells of migraineurs

**DOI:** 10.1186/s13578-016-0106-6

**Published:** 2016-06-10

**Authors:** Lei Hou, Dongjun Wan, Zhao Dong, Wenjing Tang, Xun Han, Li Li, Fei Yang, Shengyuan Yu

**Affiliations:** International Headache Center, Department of Neurology, Chinese PLA General Hospital, Fuxing Road 28, Haidian District, Beijing, 100853 China; Department of Neurology, General Hospital of Jincheng Anthracite Coal Ming Group, Jincheng, 048000 Shanxi Province China

**Keywords:** PACAP, mRNA expression, Headache, Migraine

## Abstract

**Background:**

Pituitary adenylate cyclase-activating polypeptide (PACAP) plays several important roles in vasodilation, neurotransmission, neuromodulation and neurotrophy, as well as activation of the trigeminovascular system. The aim of the present study was to explore the relationship between altered PACAP levels in peripheral blood and different types of headache.

**Methods:**

The present study enrolled 101 outpatients with headache and 35 healthy control volunteers. Blood samples were collected from the cubital vein and peripheral blood mononuclear cells (PBMCs) were separated. Total mRNA in the PBMCs was extracted and the expression of PACAP mRNA was analyzed by quantitative-polymerase chain reaction (Q-PCR).

**Results:**

There was a significant decrease in PACAP mRNA expression in the PBMCs of the migraine (M) group relative to the healthy control group. However, there were no significant differences in PACAP mRNA expression between the control group and tension-type headache (TTH), cluster headache (CH), or medication overuse headache (MOH) groups.

**Conclusion:**

The PBMC levels of patients with migraine, but not other commonly seen headache types, exhibited a significant reduction in PACAP mRNA expression compared with healthy control subjects.

## Background

Headache can be classified as primary and secondary disorder. The latter was usually caused by another disease, trauma or any certain factors such as medication overuse, tumors or inflammation, while the primary headache was independent [1]. Approximately 90 % of all headaches are primary, and the most common subtypes are migraine (M), tension-type headache (TTH), and cluster headache (CH) [1–3]. The pathophysiology of headache includes traction to or irritation of the meninges and blood vessels, because the brain itself is not sensitive to pain due to its lack of nociceptors [4]. These receptors, which are located at the extracranial arteries, middle meningeal artery, large veins, and cranial or spinal nerves, send projections up the length of the nerve fiber to nerve cells in the brain and signal that a part of the body hurts [5].

Pituitary adenylate cyclase-activating polypeptide (PACAP) is a member of the vasoactive intestinal peptide (VIP)/secretin/glucagon peptide family, which is widely expressed in humans in the central nervous system (CNS), endocrine glands, and peripheral organs [6]. Therefore, PACAP functions as a pleiotropic peptide that influences neurotransmission, neuromodulation, and neurotropic actions in the CNS, vessel dilation and regulatory mechanisms in the gastrointestinal, cardiovascular, reproductive, and respiratory systems, and anti-inflammatory activity in the immune system [7–9]. Moreover, recent studies have demonstrated an important role of PACAP in nociceptive processes [10–12], identified PACAP in the trigeminal system [12], and observed the co-localization of nociceptors and PACAP [13, 14]. Additionally, Schytz et al. [15] demonstrated that infusion of PACAP causes headache and vasodilatation in both healthy subjects and migraine patients. Taken together, these findings indicate that PACAP is involved in the pathogenesis of headache, but the exact roles it plays in different types of headache remain unclear.

Thus, the present study measured PACAP mRNA expression in peripheral blood mononuclear cells (PBMCs) of headache patients and healthy controls, and aimed to determine the relationships between altered PACAP levels in peripheral blood and different types of headache.

## Methods

### Participants

The present study assessed 101 outpatients suffering from headache that visited the International Headache Center in the Department of Neurology at Chinese People’s Liberation Army (PLA) General Hospital from April 2014 to February 2015, and 32 healthy people were recruited as a control group. Patients were selected in accordance with the criteria of the International Classification of Headache Disorders, third edition, beta version (ICHD-3 beta), and were categorized as follows: migraine (M, n = 43), tension-type headache (TTH, n = 23), cluster headache (CH, n = 13), and medication overuse headache (MOH, n = 22). The study groups were age-matched and the demographic and clinical characteristics of both patient and control populations were summarized in Table 1. A detailed questionnaire and examination were used to distinguish the type of headache in each patient, and any subjects (patients or controls) who displayed any other significant or serious disorders were excluded from the study. The study was approved by the Ethics Committee of the PLA General Hospital. All study participants signed an informed consent form, and all procedures were conducted in accordance with the Declaration of Helsinki.

**Table 1:** The demographic characteristics comparisons between different types of headaches and healthy control group

Variable	Healthy control	M	TTH	CH	MOH
Total	32	43	23	13	22
Male	14 (43.7 %)	15 (34.9 %)	7 (30.4 %)	11 (84.6 %)	3 (13.6 %)
Female	18 (56.3 %)	28 (65.1 %)	16 (69.6 %)	2 (15.4 %)	19 (86.4 %)
Age	35.46 ± 8.49	35.21 ± 10.44	45.17 ± 10.88	34.69 ± 10.12	46.18 ± 9.60
<30	9 (28.1 %)	14 (32.6 %)	2 (8.7 %)	6 (46.2 %)	1 (4.5 %)
30–40	15 (46.9 %)	12 (27.9 %)	6 (26.1 %)	4 (30.8 %)	5 (22.7 %)
>40	8 (25 %)	17 (39.5 %)	15 (65.2 %)	3 (23.1 %)	16 (72.7 %)
Education					
Junior colleague or lower	12 (37.5 %)	26 (60.4 %)	17 (74.0 %)	9 (69.2 %)	17 (77.3 %)
University or above	20 (62.5 %)	17 (39.6 %)	6 (26.0 %)	4 (30.8 %)	5 (22.7 %)
BMI					
Underweight (<18.5)	3 (9.4 %)	3 (7.0 %)	0	1 (7.7 %)	2 (9.1 %)
Normal weight (18.5 to <23)	23 (71.9 %)	24 (55.8 %)	10 (43.5 %)	5 (38.5 %)	8 (36.4 %)
Overweight (23 to <25)	4 (12.5 %)	6 (14.0 %)	5 (21.7 %)	2 (15.4 %)	6 (27.3 %)
Obese (≥25)	2 (6.2 %)	10 (23.3 %)	8 (34.8 %)	5 (38.5 %)	6 (27.3 %)

### Sample collection and preparation

There were no restrictions placed on the subjects regarding food or drink intake. All study subjects provided blood samples (3 ml per subject). Each sample was drawn from the cubital vein, collected in an ice-cold glass tube containing the anticoagulant ethylenediaminetetraacetic acid (EDTA), and centrifuged within 1 h at 4000 rpm for 5 min at 4 °C. After centrifugation, PBMCs in the cell pellets were isolated using Ficoll-Paque PLUS (GE Healthcare; Piscataway, NJ, USA) and stored at −80 °C until the further use.

### RNA extraction and reverse transcription

Using TRIpure LS reagent (Bioteke; Beijing, China), the total RNA was extracted according to the manufacturer’s protocol. Briefly, 1 ml of fresh blood was lysed with 3 ml TRIpure LS reagent. RNA was separated using chloroform, precipitated with isopropanol, and then washed with 70 % ethanol. Next, the RNA pellet was air-dried and dissolved in RNase-free water. To remove genomic DNA contamination, total RNA was incubated with RNase-free DNase, and then the purified RNA was quantified with a spectrophotometer and stored at −80 °C. First-strand cDNA synthesis was performed for each RNA sample using the Sensiscript RT kit with random hexamers used to prime cDNA synthesis.

### Quantitative-polymerase chain reaction (Q-PCR)

Primers were designed with Primer Premier 5.0 software and synthesized by the CapitalBio Corporation (Beijing, China) as follows: GAPDH: sense 5′-TGTTGCCATCAATGACCCCTT-3′; anti-sense 5′-CTCCACGACGTACTCAGCG-3′;PACAP: sense 5′-TGTTCCCGTGGCTAGTTGC-3′; anti-sense 5′-ATCTGCTTCAGTTTAGTAAGGCTCT-3′.

The mRNA expression analyses of PACAP and phosphoglyceraldehyde dehydrogenase (GAPDH) were performed by real-time PCR with SYBR Green Master Mix (Applied Biosystems; Foster City, USA). The thermocycler conditions consisted of an initial holding at 50 °C for 2 min and then at 95 °C for 10 min; this was followed by a two-step PCR program at 95 °C for 15 s and 60 °C for 60 s for 40 cycles. All data were collected and quantitatively analyzed on an ABI Prism 7500 Sequence Detection System (Applied Biosystems). The GAPDH gene served as an endogenous control to normalize for differences in the amount of total RNA in each sample. All quantities are expressed as the number of fold increase relative to that of GAPDH.

### Statistical analysis

All data were processed using SPSS 19.0. Continuous variables are presented as the mean ± standard deviation, and categorical variables as frequency counts and percentages. The normality of the data was tested with the Shapiro–Wilk test. Additionally, a correlation analysis was performed using a Student’s unpaired t test and a single-factor analysis of variance (ANOVA) to determine whether any relationships existed between the patient characteristics and PACAP levels. Finally, the levels of PACAP were compared between the headache groups and healthy controls with a single-factor analysis of variance (ANOVA). A p value <0.05 was considered to indicate statistical significance.

## Results

### The demographic characteristics comparisons between different types of headaches and healthy control group

Comparison of the demographic data among the different headache groups and the control group is shown in Table 1. The ages of the 101 headache patients ranged from 14 to 69 years (M: 14–58 years, 35.21 ± 10.44 years; TTH: 24–69 years, 45.17 ± 10.88 years; CH: 25–55 years, 34.69 ± 10.12 years; MOH: 23–63 years, 46.18 ± 9.60 years), and the ages of the 32 control subjects ranged from 20 to 51 years (35.46 ± 8.49 years). Patients were also categorized into groups based on their body mass index (BMI; underweight, normal weight, overweight, and obese) and level of education (junior colleague or lower and university or above). A correlation analysis revealed no significant correlation between PACAP mRNA levels and any patient characteristics, including age, gender, education, or BMI (p > 0.05) (Table 2).

**Table 2:** PACAP mRNA levels and patient characteristics

	n	2^−△Ct^ (×10^−3^)	p value
Gender			>0.05
Male	50	0.5333 ± 0.0829	
Female	83	0.4907 ± 0.0676	
Age			>0.05
<30	32	0.4850 ± 0.0920	
30–40	42	0.3954 ± 0.0601	
>40	59	0.6320 ± 0.0962	
Education			>0.05
Junior colleague or lower	81	0.4558 ± 0.0528	
University or above	52	0.6146 ± 0.1042	
BMI			>0.05
Underweight (<18.5)	9	0.4677 ± 0.2037	
Normal weight (18.5 to <23)	70	0.5166 ± 0.0675	
Overweight (23 to <25)	23	0.4123 ± 0.1104	
Obese (≥25)	31	0.4123 ± 0.1104	

### The PACAP expression in the PBMCs of headache patients and the healthy controls

**Fig. 1:**
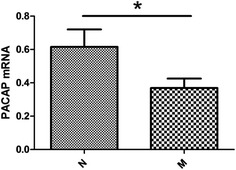
PACAP expression in migraine groups and controls. Comparison of −ΔCt of PACAP mRNA expression between migraine patients and the control groups showed that control group had higher −ΔCt than the patient group (controls: n = 35; mean −ΔCt: 0.6155 ± 0.1050; migraineurs: n = 43; 0.3693 ± 0.0562; p = 0.0304)

**Fig. 2:**
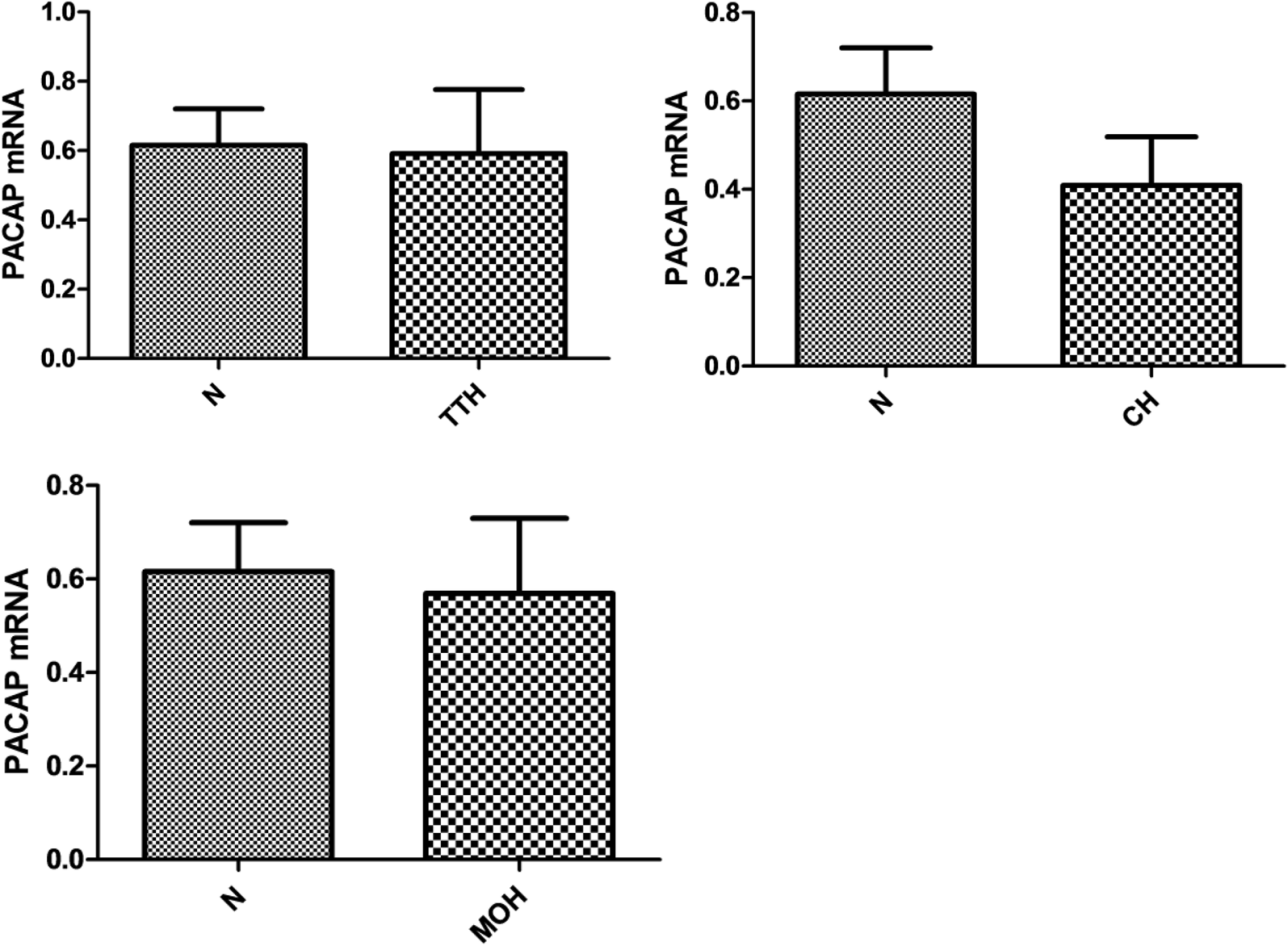
PACAP expression in CH, TTH, MOH groups and controls. Comparison of −ΔCt of PACAP mRNA expression between TTH, CH and MOH patients and the control groups showed no significant differences between the patient groups and controls.(controls: mean −ΔCt: 0.6155 ± 0.1050; TTH: 0.4683 ± 0.1448, p = 0.4035; CH: 0.4092 ± 0.1099, p = 0.2570; MOH: 0.5371 ± 0.1785, p = 0.8014)

To determine the expression of PACAP mRNA in the PBMCs of headache patients, PBMCs were isolated and then evaluated using Q-PCR. To examine whether the level of PACAP mRNA expression in the PBMCs correlated with headache, the data for M, TTH, CH and MOH groups were compared with the healthy control group respectively. Figure 1 shows that compared with control groups (n = 32; 0.6155 ± 0.1050), there was an obvious decrease in the level of PACAP expression in the PBMCs of the M group (n = 43; 0.3693 ± 0.0562; p = 0.0304). However, no significant difference was observed between the TTH, CH, MOH groups and control groups (p > 0.05) (Fig. 2), which suggested relative specificity for migraine.

### The PACAP expression in the PBMCs of migraineurs

**Fig. 3:**
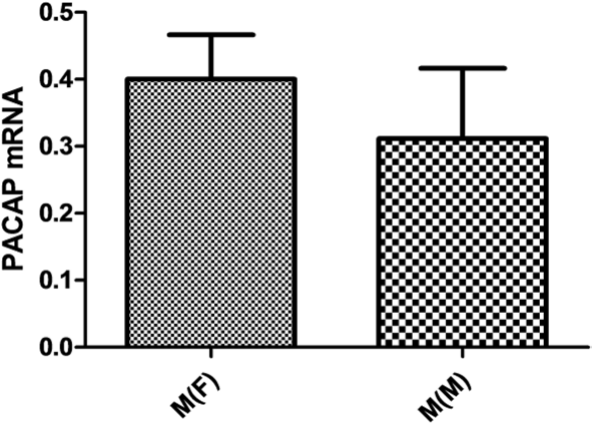
PACAP expression in female and male migraineurs. No significant differences of PACAP expression in PBMCs of patients were observed between male and female migraineurs. (female: mean −ΔCt 0.4003 ± 0.06623; male: 0.3114 ± 0.1052, p > 0.05)

**Fig. 4:**
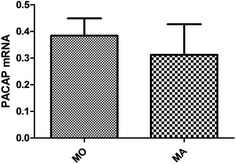
PACAP expression in migraine with (MA) and without aura (MO). No significant differences of PACAP expression in PBMCs of patients were observed between MA and MO groups. (MO: mean −ΔCt 0.3843 ± 0.06496; MA: 0.3125 ± 0.1146, p > 0.05)

**Fig. 5:**
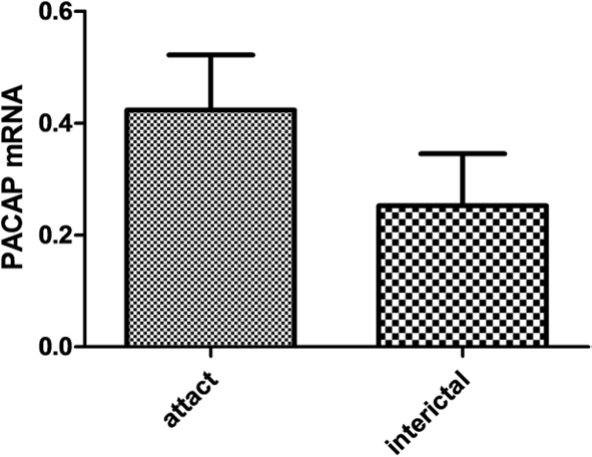
PACAP expression in migraine attack and interictal groups. The results presents an uptrend of PACAP expression during migraine attack compared with interictal (attack groups: mean −ΔCt 0.3994 ± 0.3673; interictal groups: 0.2923 ± 0.3560, p > 0.05)

The results above suggested the PACAP expression in the PBMCs has correlation with migraine, but not with TTH, CH and MOH. Therefore, to investigate the relationship between the PACAP expression and migraineurs’ characteristics, the migraine group was subdivided into male and female groups, interictal and attack groups, and with aura (MA) and without aura (MO) groups, but no significant differences in PACAP levels were observed (p > 0.05) (Figs. 3, 4, 5). But Fig. 5 presents an uptrend of PACAP expression during migraine attack compared with interictal (0.3994 ± 0.3673 vs 0.2923 ± 0.3560).

## Discussion

The present findings identified a relationship between the expression of PACAP mRNA in mononuclear cells derived from the peripheral blood of headache patients and different types of headache. A detailed analysis revealed that the PBMCs of migraineurs exhibited a significantly lower level of PACAP mRNA expression than that of the control group, but there were no significant differences between the control group and TTH, CH, or MOH groups. Moreover, no significant correlation was observed between PACAP expression and age, gender, BMI, or the pain intensity and duration of headache in any headache group. These findings suggest that PACAP may be an effective marker for the identification of migraineurs and that the monitoring of these levels can inform a physician about the progression of migraine.

PACAP is a member of the VIP/secretin/glucagon peptide family encoded by the ADCYAP1 gene and is widely expressed in the CNS and peripheral tissues [16]. It has been reported that PACAP can induce neurogenic inflammation, neuronal activation and sensitization, and mast cell degranulation, which can all be involved in headache development [17]. Moreover, a previous study from our research group found that patients suffering from migraine exhibited lower plasma levels of PACAP compared to healthy control donors. Additionally, a negative correlation between interictal PACAP-38-LI and the duration of migraine was recently shown, but no relationships between plasma PACAP levels and gender, age, attack frequency, allodynia, or hormonal changes have been demonstrated [15]. Furthermore, although in the present study, no significant differences in PACAP expression were found between the control group and TTH, CH, or MOH groups, the PACAP expression in migraineurs was significantly higher than that in the control group, which indicate that PACAP may act as a potential marker highly specific for migraine.

However, which types of cells that act as the major source of or cause the changes in PACAP levels in patients with migraine remain unclear. Migraine has been associated with disordered immune responses [18] and PACAP protein can be secreted by multiple types of cells, including neurons and immune cells [6]. Thus, the detection of PACAP expression in immune cells may explain the correlation between immune responses and migraine. Isolation of PBMCs in the present study revealed that migraineurs exhibited a lower level of PACAP mRNA than the healthy controls. This is in accordance with the results of a previous study from our research group which indicated that the level of PACAP mRNA in PBMCs reflects changes in plasma levels of PACAP [19]. However, in the present study, there was no relationship between the levels of PACAP mRNA in PBMCs and age, gender, BMI, or the pain intensity and duration of migraine. Further investigation is warranted to determine whether other peripheral tissue cells with similar patterns of PACAP expression can be described in the plasma and PBMCs.

The present study also assessed PACAP mRNA expression in the PBMCs of patients suffering from TTH, CH and MOH. In contrast to migraineurs, no significant differences in PACAP mRNA expression were found compared to the healthy control group, indicating that the PACAP mRNA level in PBMCs may be used as a marker for the differential diagnosis of migraine. To date, other than the calcitonin gene-related peptide (CGRP) [20], no markers has been identified,which could classify the headache into different subtypes.

In the present study, monitoring of PACAP mRNA expression in PBMCs during the progression of M revealed that PACAP levels were higher in the attack stage than the interictal stage. It was previously shown that migraineurs have elevated PACAP levels during the ictal period relative to the attack-free period [21]. Taken together, these findings suggest that PACAP may be involved in the development of migraine attacks and that the difference between these two stages may be due to different types of PACAP and various levels of protein and mRNA expression. Future studies should include a larger number of patients and perform detailed analyses investigating PACAP protein and mRNA levels simultaneously.

The findings of the present study indicate that PACAP mRNA levels in PBMCs may be used to conduct a differential diagnosis of migraine. Additionally, the changes in PACAP levels in PBMCs may represent a potential mechanism that links immune responses and the CNS.

## Conclusion

The PBMC levels of patients with migraine, but not other commonly seen headache types, exhibited a significant reduction in PACAP mRNA expression compared with healthy control subjects. The findings indicate that PACAP mRNA levels in PBMCs may be used to conduct a differential diagnosis of migraine and the changes in PACAP levels in PBMCs may represent a potential mechanism that links immune responses and the CNS.
